# Sex comparisons across different indices of vascular health in recreationally active healthy adults

**DOI:** 10.1007/s00421-026-06335-1

**Published:** 2026-07-03

**Authors:** Rian Q. Landers-Ramos, Olivia C. Zakrewski

**Affiliations:** Department of Kinesiology, Towson University, Towson, MD 21252 USA

**Keywords:** Vascular health, Sex difference, Arterial stiffness, Microvascular reactivity

## Abstract

**Introduction:**

The presence of sex differences in various vascular assessments in younger to middle-aged adults is inconsistent in the literature.

**Purpose:**

Determine whether sex differences are present across four different indices of vascular health.

**Methods:**

Healthy, recreationally active premenopausal females (*N* = 30) and similarly aged males (*N* = 30) aged 18–54 yrs performed four vascular assessments. Assessments included resting augmentation index (AIx%), carotid-femoral pulse wave velocity (cf-PWV, ms^− 1^), and simultaneous digital thermal monitoring (temperature rebound; TR%) and near-infrared spectroscopy-based microvascular reactivity (10s reperfusion slope; slope 2, %s^− 1^).

**Results:**

AIx (*p* = 0.006) was significantly lower in males vs. females. Sex differences were eliminated after covarying for mean adjusted height (8.9 ± 9.1% vs. 16.8 ± 11.9% in males and females, respectively; *p* = 0.360) or when examining a subset of individuals (*N* = 14 males and *N* = 14 females) with similar body height (13.0 ± 8.7% vs. 16.4 ± 7.9% in males and females, respectively; *p* = 0.286). Cf-PWV was significantly higher in males (6.0 ± 0.8 ms^− 1^) vs. females (5.2 ± 0.7 ms^− 1^; *p* < 0.001). We found no differences between males and females in TR (1.2 ± 1.1% vs. 1.1 ± 0.8%, respectively; *p* = 0.771), or StO_2_ slope 2 (2.2 ± 0.6%s^− 1^ vs. 1.9 ± 0.9%s^− 1^, respectively; *p* = 0.254).

**Conclusions:**

After accounting for confounding variables, differences in cf-PWV were detected between sexes, while sex differences in other assessments of vascular health were not observed.

## Introduction

Premenopausal females typically exhibit reduced cardiovascular disease (CVD) risk compared with age-matched males (Benjamin et al. 2018; Stanhewicz et al. 2018; Kim et al. 2025). This is potentially attributable to more favorable vascular health, including large blood vessel endothelial function (Stanhewicz et al. 2018), arterial stiffness (Doonan et al. 2013), and microvascular function (Molbo et al. 2022). However, the presence of sex differences in recreationally active younger and middle-aged adults across various vascular assessments is inconsistent in the literature, warranting more work in this area (Waddell et al. 2001; Tomiyama et al. 2003; McEniery et al. 2005; Doonan et al. 2013; Naghavi et al. 2016; Keller et al. 2023; Landers-Ramos et al. 2024a, b; Murias et al. 2026).

Augmentation index (AIx) refers to the pressures imposed on the left ventricle from systolic peripheral wave reflection (Kim and Braam 2013; Heusinkveld et al. 2019). Resting AIx is reportedly higher in females (McEniery et al. 2005). However, shorter stature in females leads to earlier pressure wave reflection and therefore higher AIx (Doonan et al. 2013). We (Landers-Ramos et al. 2022) and others (Lieber et al. 2010; Shim et al. 2011; Doonan et al. 2013) have previously shown that sex differences in AIx are eliminated after controlling for body height. However, this has not been reported consistently, warranting more research in this area (Gatzka et al. 2001; Ayer et al. 2010; Zaniqueli et al. 2018). While frequently not reported, analysis of the different components of wave reflection (forward and backward pressure waveforms) may provide a greater understanding of the influence of body height on wave reflection compared with AIx alone (Mitchell et al. 2004; Li et al. 2017). Further, carotid-femoral pulse wave velocity (cf-PWV) is considered the reference standard for assessing arterial stiffness and is calculated using the carotid-femoral path length influenced by body height. Thus, inclusion of cf-PWV can help with the interpretation and conclusions generated from wave reflection analyses. Collectively, considering cf-PWV, AIx, and related pressure wave reflections together will allow a better collective understanding of the impact of biological sex on arterial stiffness in younger and middle-aged adults.

Associations between arterial stiffness and CVD are mediated by microvascular function (Cooper et al. 2016). Dysfunction in the microvasculature can manifest at earlier ages than can be detected in the macrovasculature (Limberg et al. 2020), providing valuable prognostic information in younger and middle-aged adults. Challenges in assessing microvascular function and varying methodologies have led to inconsistent findings regarding sex differences (Limberg et al. 2020; Zocalo and Bia 2021; Molbo et al. 2022; Landers-Ramos et al. 2024a; Fekete et al. 2025; Murias et al. 2026). Several studies have reported sex differences in near-infrared spectroscopy (NIRS)-derived skeletal muscle microvascular reactivity (Rasica et al. 2022; Keller et al. 2023; Citherlet et al. 2024; Murias et al. 2026), whereas we recently found that these differences were no longer apparent after accounting for adipose tissue thickness (ATT) underlying the NIRS probe (Landers-Ramos et al. 2024a). This study was one of few to address the role of ATT in NIRS-based microvascular assessments and only considered one muscle (the tibialis anterior). Thus, further research in this area is warranted to examine whether sex differences in other skeletal muscle microvascular responses are present when accounting for factors affecting the NIRS signals.

Relationships between NIRS-derived reperfusion and flow-mediated dilation or blood flow velocity (macrovascular function) have been reported (McLay et al. 2016; Murias et al. 2026), but not consistently (Matthews et al. 2021). However, there is a need for additional studies examining the relationship between NIRS-derived microvascular reactivity and other microvascular assessments. Digital thermal monitoring (DTM) serves as a measure of peripheral vasodilation (Gul et al. 2009; Heath et al. 2021) and can be performed alongside NIRS to allow for concurrent measures of microvascular reactivity. Simultaneous measures may provide greater insight into the consistency of any observed sex differences in microvascular function.

Collectively, there is a need for further work examining the effects of biological sex on multi-component assessments of vascular health. The purpose of this study was to determine whether sex differences are present across four different indices of vascular health in premenopausal females and similarly aged males. We hypothesized that when appropriately accounting for confounding variables, there would be no sex differences in AIx, cf-PWV, NIRS-derived microvascular reactivity, and DTM-based peripheral vasodilation. As part of an exploratory aim conducted in a subset of individuals, we hypothesized that there would be a significant positive relationship between NIRS-derived microvascular reactivity and peripheral vasodilation measured via DTM.

## Methods

### Participants and study design

This study was approved by the Towson University Institutional Review Board (#2341). Written informed consent was obtained from all participants, and all study procedures adhered to those outlined in the Declaration of Helsinki. All participants provided consent to publish. Participants were healthy males and females between the ages of 18 and 55 years and were recruited from the Baltimore County, Maryland area. Participants were recreationally active, defined as participating in 30 min/day of moderate-intensity cardiovascular and/or resistance training on 3–5 days/week. Recreationally active individuals were selected to best compare our major outcomes to previous work investigating participants with similar physical activity levels (Doonan et al. 2013; Landers-Ramos et al. 2022, 2024a; Keller et al. 2023; McGranahan et al. 2023; Zaleski et al. 2023; Jeskanen et al. 2024; Perlet et al. 2024; Murias et al. 2026). Self-reported physical activity was determined using an abbreviated International Physical Activity Questionnaire (Bassett 2003). Briefly, participants were asked to quantify the number of minutes per week they spent performing resistance training (RT), aerobic exercise, and recreational sports in the 7 days prior to their study visit. Inactive (< 20 min of structured exercise/day on > 2 days/week) and trained or competitive athletes were excluded from this study due to the potential contribution of training status to our study outcomes. Participants were excluded if they had a body mass index (BMI) > 30 kg/m^2^, were regular smokers (within the past 6 months), or had previously diagnosed coronary heart disease, congenital heart disease, or circulation conditions. Post menopausal females, defined as the absence of a menstrual cycle for > 1 year, were also excluded from this study. Amenorrheic females were also excluded from this study. Menstrual cycle phase and oral contraceptive use have been found to have minimal effects on vascular outcomes (Priest et al. 2018; Shenouda et al. 2018; Mattu et al. 2020; Williams et al. 2020; D’Souza et al. 2023), and so these were not controlled for in this study. Rather, the menstrual cycle phase was estimated based on participants’ self-reports and documented.

### Study design

The study took place in a single visit to the laboratory. Participants arrive fasted (no food, caffeine, medications, or alcohol) for approximately 12 h before their visit. Participants also abstained from moderate-to-high intensity exercise for approximately 24 h before their visit. Upon arrival, baseline assessments (height, weight, blood pressure, body composition) were performed in the morning between 6:00 and 11:00 AM. Height (stadiometer, SECA North America, Chino, CA, USA) and weight (digital scale, Tanita Corporation, Arlington Heights, IL, USA) were measured, and BMI was calculated as body weight in kg/body height in m^2^. Seated heart rate (HR) measurements and brachial blood pressure BP were measured on the participant’s dominant arm with an automated device (Omron, Kyoto, Japan). Participants then completed a dual-energy X-ray absorptiometry (DXA) scan followed by four vascular assessments. Details of each measurement are outlined below.

### Dual-energy X-ray absorptiometry

Body composition was measured using a DXA scan (Lunar Prodigy Z-ray Bone Densitometer, GE Healthcare, Chicago, IL, USA), as done previously (Zabriskie et al. 2020; Landers-Ramos et al. 2022). Participants were positioned on the DXA table by trained members of the research team. Quality assurance measures were performed daily (average CV over the study period 3.1%). All scans were analyzed using Encore software (version 14.0) according to the manufacturer. Total body fat mass, lean mass, and bone mass were measured. Percent body fat was recorded for all participants. Visceral adipose tissue (VAT) was estimated using the manufacturer’s CoreScan algorithm, which provides VAT volume derived from abdominal fat distribution using a proprietary model (Shepherd et al. 2017).

### Augmentation index and wave reflection

Participants rested in the supine position for 10 min before central blood pressure was derived with an automated device employing specialized cuff-based brachial waveform analysis (SphygmoCor, AtCor Medical, Sydney, NSW, Australia). Aortic pressure waveforms were reconstructed from the brachial artery pressure waveforms using a generalized validated transfer function (Hwang et al. 2014). AIx was defined as the difference between the first and second systolic peaks divided by pulse pressure. AIx is expressed as a percentage and normalized to a heart rate of 75 bpm (AIx75) (Wilkinson et al. 2002). Forward (Pf) and reflected (Pb) pressure waveforms were also computed using wave separation analyses performed with the SphygmoCor Cardiovascular Suite software (SphygmoCor, AtCor Medical Sydney, NSW, Australia). Pf represents the heart-generated pressure wave traveling into the periphery. Pb represents the reflection of the initial wave from the periphery back to the heart (Torjesen et al. 2014). Reflection magnitude was calculated and reported as the ratio of Pb to Pf magnitude and expressed as a percentage [Reflection Magnitude = Pb/Pf x 100]. The Sphygmocor Xcel system has been found to produce valid and reliable measurements of augmentation index and wave reflection (Hwang et al. 2014).

As body height is known to influence AIx, a subset of males and females of similar body height was analyzed from the complete cohort of participants. Specifically, *N* = 14 males and *N* = 14 females with body heights between 1.63 m and 1.76 m were selected to determine whether sex differences in AIx and wave reflection were evident when body height was similar between sexes.

### Central arterial stiffness

Cf-PWV was performed using cuff-based applanation tonometry (SphygmoCor, AtCor Medical Sydney, NSW, Australia) to obtain pressure waveforms from the femoral artery, while carotid pressure waveforms were obtained using hand-held tonometry. A standard tape measure was used to measure distances from the sternal notch to the carotid and femoral artery sites and from the location on the femoral artery where the pulse was detected to the top of the blood pressure cuff located on the upper thigh. All measurements were performed on the right side of the body. These distances were used to determine the path length for cf-PWV. Waveforms were collected using a high-fidelity tonometer over a 10-second capture period. Cf-PWV was calculated as Δ distance (m)/Δ time (s), where Δ time is the time delay between pulse arrivals at the carotid and femoral arteries.

### Digital thermal monitoring

**Fig. 1:**
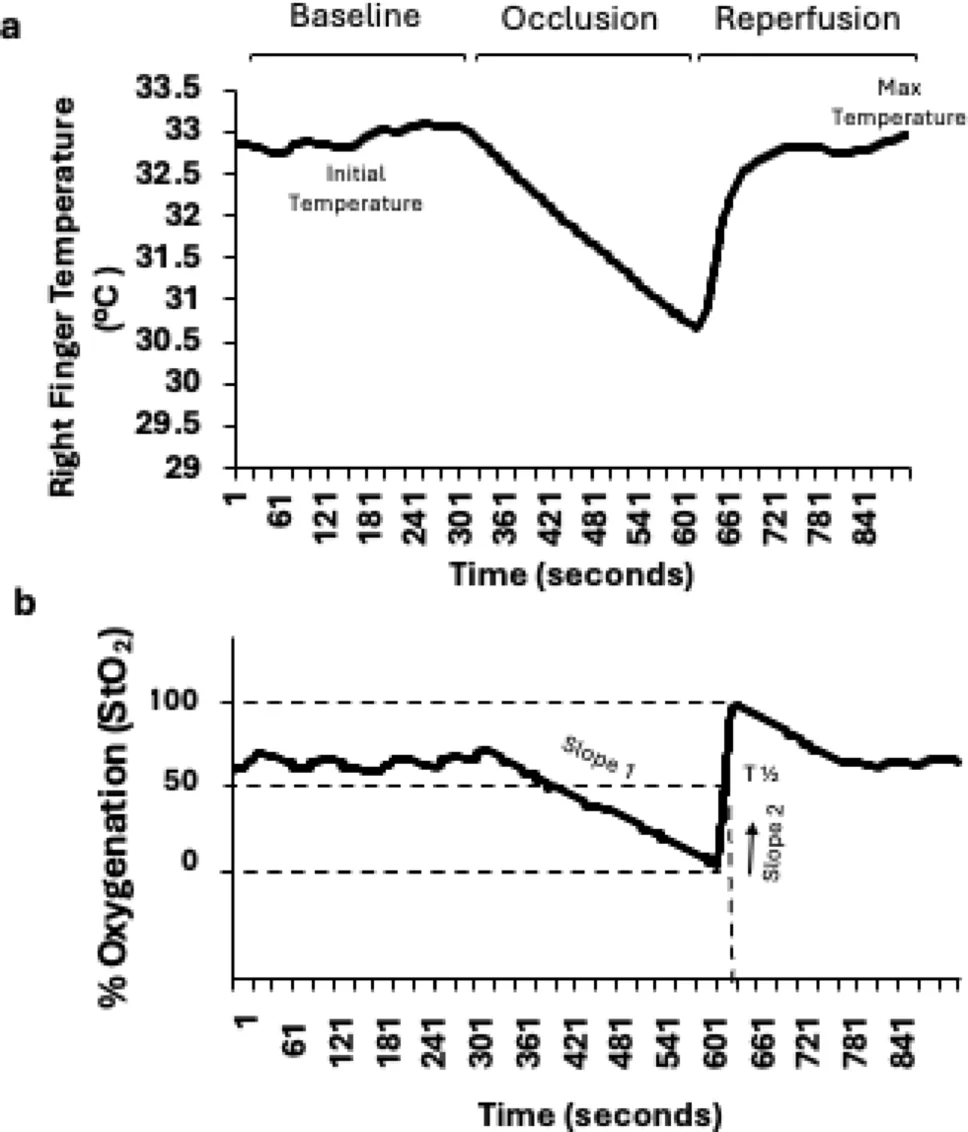
Representative tracings of the **a** digital thermal monitoring and **b** StO_2_ signal during the vascular occlusion test. Tests were performed simultaneously and consisted of five minutes of baseline testing, 5 min of limb occlusion, and 5 min of reperfusion

DTM was used to assess peripheral vasodilation (Akhtar et al. 2010; Schier et al. 2014). The participants lay supine with a warming pad placed over their shoulders for 20 min before testing to maintain a body temperature above 27 °C. Thermocouple fingertip probes were affixed to the index finger on each hand and were connected to a VENDYS (Endothelix, Inc., Houston, TX, USA) system. A blood pressure cuff was placed on the right upper arm, and blood pressure was recorded. The cuff was then replaced with a cuff (Delfi Medical Innovations, Vancouver, BC) capable of rapid inflation and maintaining pressure throughout an extended period without the need for reinflation. A vascular occlusion test (VOT) was performed and included 5 min of baseline measures, followed by a 5-min occlusion at a pressure of ≥ 50 mmHg above systolic blood pressure (Fig. 1a). The cuff was then rapidly deflated, and data on reperfusion and finger temperature recovery were collected for 5 min. Thermal tracings in the right and left finger temperature before, during, and after occlusion were automatically recorded using a computer-based thermometry system with 0.006 °C thermal resolution at a sampling rate of 1 Hz. Post-deflation temperature rebound of the right finger was recorded, and temperature rebound (TR) was calculated as (max temperature – initial temperature/initial temperature x 100). Using a proprietary method, VENDYS software also calculated adjusted temperature rebound [(aTR (A.U.)] as an indicator of vascular reactivity (Ahmadi et al. 2009; Mcquilkin et al. 2009; Akhtar et al. 2010; Schier et al. 2013, 2014; Karimzad et al. 2023). The aTR calculation assumes a fingertip temperature rebound curve if no vascular reactivity were present as “zero reactivity curve”, and accounts for baseline finger temperature and the actual observed degree of temperature fall during the 5-min cuff occlusion (VENDYS II Manual, Endothelix, Inc., Houston, TX, USA). The temperature of the left finger was recorded as a control for sympathetic responses to the occlusion. Tests that were flagged by the system for cold fingers (< 27 °C) or sympathetic response (decline in left finger temperature > 0.5 °C after right arm occlusion) were excluded from analyses. The room was quiet and dim, and the room temperature was kept at approximately 24 °C for the length of the test. Tests in which the room temperature and baseline finger temperature were < 3 °C different were also discarded.

### Skeletal muscle microvascular reactivity

Microvascular reactivity of the forearm muscle was performed using a NIRS-based VOT (Fig. 1b). The length of the forearm was measured from the medial epicondyle of the humerus to the styloid process of the ulna using a standard tape measure. The area 1/3 of the distance from the proximal location was marked on the belly of the forearm. A high-resolution ultrasound (GE Logiq, GE Healthcare Products, Chicago, IL, USA) equipped with a 7.5–12 MHz linear array transducer was used to determine ATT and muscle thickness (MT) of all forearm flexor muscles below the marked area. The area was then cleaned, and the NIRS device (Portalite, Artinis Medical Systems, Elst. Netherlands) was secured over the marked location on the forearm muscle using opaque athletic foam wrap to prevent ambient light from interfering with the NIRS signal. Participants were asked to lie supine on a table with their arms placed comfortably by their side. A tapered 4” x 18” blood pressure cuff (Delfi Medical Innovations, Vancouver, BC) was placed on the upper arm. The cuff was connected to a rapid inflation system (Hokanson E2, Hokanson, Inc., Carmel, IN, USA) and a high-capacity air source (AG101, Hokanson, Inc., Carmel, IN, USA), which allows for inflation of the cuff in ~1s. To aid in participant comfort and to reduce movement due to initial responses to unfamiliar stimuli, participants were familiarized with rapid cuff inflation before testing. This test was performed simultaneously with the DTM test described above, so times and cuff pressures were identical. Participants were asked to remain still, and NIRS data were collected continuously throughout the test. NIRS-derived data were exported as 10 Hz files, and analyses were performed with a custom Python code using the tissue oxygen saturation (StO_2_) signal (calculated as [O_2_Hb/(O_2_Hb + HHb)]). StO_2_ was used for all analyses during the VOT as previously reported (McLay et al. 2016; Caldwell et al. 2023; Landers-Ramos et al. 2024a; Murias et al. 2026). Baseline StO_2_ (%) was calculated as the average StO_2_ for 1 min before the start of occlusion. StO_2_ minimum (min; %) was defined as the lowest value recorded during occlusion, and the StO_2_ maximum (max; %) was defined as the highest value detected after cuff release. The min and max signals were used to determine the physiological range of the StO_2_ signal, which is reported as O.D. units. The desaturation slope (slope 1), representing muscle oxygen consumption (mVO_2_), was defined as the steepest 30 s segment during the 5-minute occlusion (% s^− 1^) (Perlet et al. 2024; Landers-Ramos et al. 2025a). Desaturation magnitude was calculated as the difference between baseline StO_2_ and the StO_2_ min (Murias et al. 2026). NIRS-based forearm muscle microvascular reactivity was quantified using two methods. First, the reperfusion slope (slope 2) was defined as the 10 s upslope immediately after cuff release (% s^− 1^) (McLay et al. 2016; Iannetta et al. 2019; Landers-Ramos et al. 2024a). Second, halftime to reperfusion (T ½) was defined as the time in seconds for the StO_2_ signal to increase from the minimum to half the maximum signal after cuff release (Willingham et al. 2016; McGranahan et al. 2023; Landers-Ramos et al. 2024a, 2025b).

### Statistical analysis

Sample size calculations for examination of sex differences yielding 80% power at an α level of 0.05 were performed using effect sizes estimated from previous published work examining between-subjects differences in each major outcome measure. Calculations for AIx indicated a sample size of 9–23 per group (Doonan et al. 2013; Kroker-Lobos et al. 2020; Landers-Ramos et al. 2022), cf-PWV indicated 20–30 per group (Doonan et al. 2013; Nieman et al. 2013), DTM indicated between 4 and 23 per group (Ahmadi et al. 2009; Gul et al. 2009; Wang et al. 2025), and StO_2_ slope 2 (Rasica et al. 2022; Keller et al. 2023; Zaleski et al. 2023) indicate a sample size between 8 and 14 per group. Thus, a sample size of 30 per group was obtained as a conservative estimate to yield enough power to detect significant differences between males and females.

Assumptions of normality and sphericity were verified for all outcome measures. Independent *t*-tests were used to assess sex differences in subject characteristics and vascular outcomes. Pearson correlation coefficients were also performed between our major dependent variables and other potentially influencing variables that differed between sexes (body height, resting HR, min/wk of RT, body fat %) (Doonan et al. 2013; Landers-Ramos et al. 2022) as well as factors previously established to impact NIRS results (StO_2_ desaturation magnitude, StO_2_ desaturation rate, ATT, MT) (Landers-Ramos et al. 2024a; Murias et al. 2026), or vascular function (age). Factors that significantly correlated with our major outcome variables were further assessed in a multivariable regression. Based on the regression model, variables found to be significant predictors of each major dependent variable were included in an analysis of covariance (ANCOVA), and the partial eta squared (η_p_^2^) for each covariate was reported in our results. Pearson correlation coefficients were also performed to determine whether there was a relationship between different vascular assessments, in particular, PWV and AIx, and simultaneously tested vascular assessments (NIRS and DTM-derived outcomes). Effect sizes using Hedge’s g were calculated for all statistically significant comparisons. For these results, the effect was determined trivial if < 0.2, small if the effect size was between 0.2 and 0.5; medium if between 0.5 and 0.8, large if > 0.8 (Cohen 1988). All data analyses were performed using SPSS v.25 (SPSS, Inc. Chicago, IL). All data are presented as the mean ± SD unless otherwise specified. Statistical significance was accepted at a *p*-value of ≤ 0.05.

## Results

### Participant characteristics

Subject Characteristics can be found in Table 1. A total of 60 individuals completed the study (*N* = 30 males). Self-reported aerobic exercise was similar between males (302 ± 226 min/wk) and females (293 ± 248 min/wk; *p* = 0.882). Recreational exercise was also similar between males (45 ± 113 min/wk) and females (61 ± 151; *p* = 0.640). Self-reported RT was significantly higher in males (249 ± 190 min/wk) compared with females (103 ± 100 min/wk; *p* < 0.001). Of the female participants, *N* = 1 reported using an intrauterine device, and *N* = 6 reported taking pharmacological contraceptives. *N* = 16 and *N* = 14 females reported menstrual cycles in the luteal and follicular phases, respectively.

**Table 1:** Participant characteristics

	Male (*N* = 30)	Female (*N* = 30)	*p*-value (Hedge’s g)
Age (yrs)	28 ± 10 (Range 18–49)	29 ± 12 (Range 18–52)	0.893 (0.090)
Seated resting HR (bpm)	64 ± 13	71 ± 12	**0.031 (0.559)**
Seated brachial SBP (mmHg)	122 ± 15	116 ± 10	0.060 (0.875)
Seated brachial DBP (mmHg)	72 ± 13	72 ± 8	0.903 (0.248)
BMI (kg/m ^2^)	26 ± 3	24 ± 3	0.103 (0.667)
Body fat (%)	20 ± 6	31 ± 7	**< 0.001 (1.69)**
VAT (cm ^3^)	369 ± 385	222 ± 209	0.079 (0.477)
ATT (cm)	0.26 ± 0.10	0.46 ± 0.23	**< 0.001 (1.12)**
MT (cm)	4.44 ± 0.55	3.49 ± 0.45	**< 0.001 (1.89)**

Means ± SD

*HR* heart rate, *SBP* systolic blood pressure, *DBP* diastolic blood pressure, *BMI* body mass index, *VAT* visceral adipose tissue, *ATT* adipose tissue thickness, *MT* muscle thickness

### Brachial and central blood pressures

Supine brachial SBP was significantly lower in females (114 ± 11 mmHg) compared with males (127 ± 18 mmHg; *p* = 0.001, *g* = 0.875), while supine brachial DBP was similar between sexes (70 ± 7 mmHg vs. 68 ± 7 mmHg in males and females, respectively; *p* = 0.473, *g* = 0.248). Aortic SBP was significantly lower in females (102 ± 10 mmHg) compared with males (109 ± 6 mmHg, *p* = 0.003, *g* = 0.852), and aortic DBP was similar between males (71 ± 7 mmHg) and females (69 ± 9 mmHg, *p* = 0.270, *g* = 0.24). Mean arterial pressure (MAP) was not significantly different between sexes (84 ± 7 vs. 82 ± 9 mmHg in males vs. females, respectively; *p* = 0.274, *g* = 0.288). Pulse pressure (PP) was significantly higher in males (37 ± 7 mmHg) vs. females (33 ± 5 mmHg; *p* = 0.004, *g* = 0.786), and augmentation pressure (AP) was significantly lower in males (3.2 ± 3.3 mmHg) vs. females (5.8 ± 4.6 mmHg; *p* = 0.015, *g* = 0.653). PP was correlated with body height (*r* = 0.258, *p* = 0.048) and resting HR (*r* = −0.344, *p* = 0.008), but regression analyses indicated that only resting HR (*p* = 0.006) was a significant predictor of PP. After covarying for resting HR (η_p_^2^ = 0.069), sex differences in PP remained (*p* = 0.023). Body height (*r* = −0.379, *p* = 0.003) and body fat (*r* = 0.273, *p* = 0.036), and age (*r* = 0.257, *p* = 0.049) were correlated with AP, but regression analyses indicated that only body height was a significant predictor of AP (*p* = 0.005). When included as a covariate, sex differences in AP were no longer apparent (*p* = 0.565).

### Augmentation index and wave reflection

**Fig. 2:**
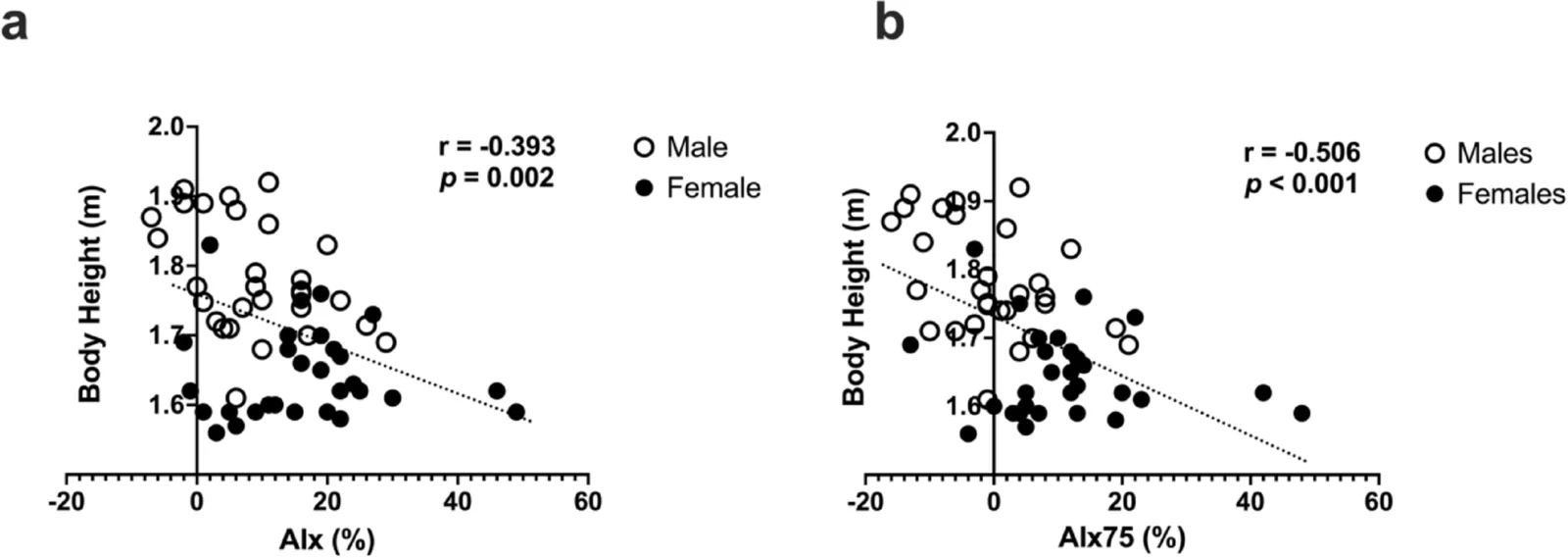
Body height is significantly correlated with **a** AIx (*p* = 0.002) and **b** AIx75 (*p* < 0.001) and was the main predictor of both AIx and AIx75 in our linear regression model

**Fig. 3:**
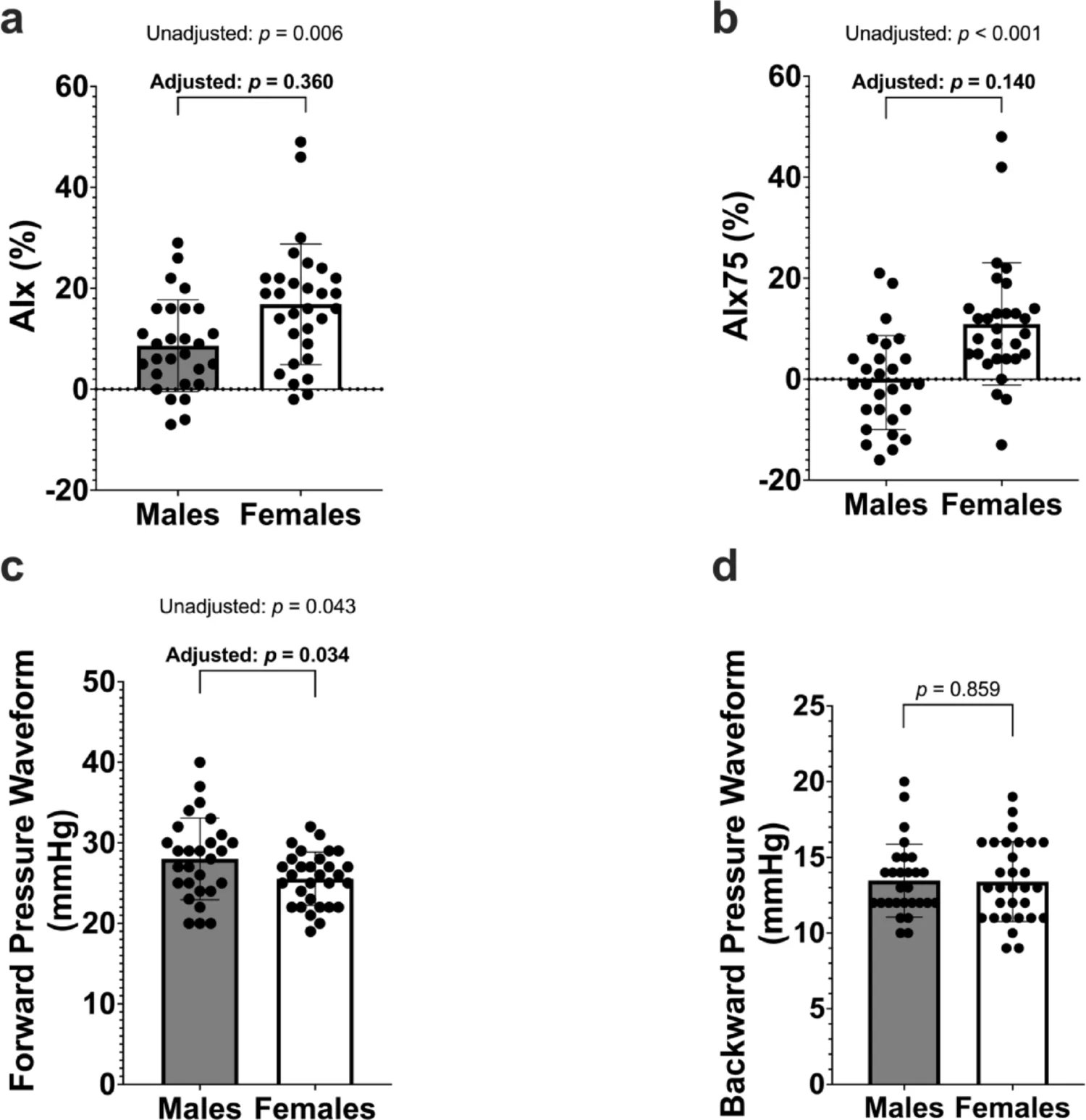
Pulse wave analysis was assessed in *N* = 30 males and *N* = 29 females. **a** Augmentation index and **b** augmentation index corrected for a heart rate of 75 bpm are presented as unadjusted and as adjusted for body height. AIx (*p* = 0.006) and AIx75 (*p* < 0.001) were both initially higher in females, but differences were eliminated after covarying for body height (*p* = 0.360 and *p* = 0.140, respectively). **c** Forward pressure waveforms were higher in males (*p* = 0.043) and remained higher after covarying for age (*p* = 0.034), and **d** reflected pressure waveforms were not significantly different between sexes (*p* = 0.859)

Pulse wave analysis was not reliably obtained from one individual, so results are presented from *N* = 30 males and *N* = 29 females. AIx (*p* = 0.006, *g* = 0.744) and AIx75 (*p* < 0.001, *g* = 0.972) were significantly higher in females vs. males. AIx and AIx75 were significantly correlated with total body fat (*r* = 0.288, *p* = 0.027, and *r* = 0.385, *p* = 0.003, respectively) and mean adjusted body height (*r* = −0.393, *p* = 0.002, and *r* = −0.506, *p* < 0.001, respectively). Age was not correlated with AIx (*r* = 0.249, *p* = 0.057) or AIx75 (*r* = 0.158, *p* = 0.233). Linear regression indicated that only mean adjusted height was a significant predictor of AIx (*p* = 0.002) and AIx75 (*p* < 0.001; Fig. 2a, b). When mean adjusted height was included as a covariate, there were no significant differences between sexes for either AIx (*p* = 0.360; Figure 3a) or AIx75 (*p* = 0.140; Figure 3b).

Pf was significantly higher in males (*p* = 0.043, *d* = 0.533, Fig. 3c), but there were no significant differences between sexes in Pb (*p* = 0.859, *d* = 0.039; Fig. 3d). Reflection magnitude was not significantly different between males (49.2 ± 7.2%) and females (52.3 ± 6.7%; *p* = 0.094, *d* = 0.445). As forward and backward pressure waveforms influence AIx, we also examined the relationships between these variables, age, body height, and body fat. There were no significant relationships between body height and Pf (*r* = 0.207, *p* = 0.116) or Pb (*r* = −0.054, *p* = 0.686) or between body fat and Pf (*r* = −0.139, *p* = 0.292) or Pb (*r* = 0.077, *p* = 0.562). Age was significantly correlated with Pf (*r* = −0.325, *p* = 0.12), but not Pb (*r* = −0.049, *p* = 0.711). When age was included as a covariate (η_p_^2^ = 0.114), sex differences in Pf remained (*p* = 0.034).

We analyzed a subset of *N* = 28 individuals (*N* = 14 males and *N* = 14 females) with similar body height (*p* = 0.088; Table 2) to further assess the contributions of body height on AIx. Participant characteristics in the subset reflected those of the full cohort except for resting HR, which was not different in the subset (*p* = 0.496). This difference is accounted for by considering both AIx and AIx75 outcomes. We found no significant differences in AIx (*p* = 0.286, *g* = 0.411) or AIx75 (*p* = 0.132, *g* = 0.588) in the groups with similar body heights. Additionally, forward pressure waveform (*p* = 0.244, *g* = 0.452), backward pressure waveform (*p* = 0.469, *g* = 0.276), and reflection magnitude (*p* = 0.857, *g* = 0.075) were not different between sexes in the subset with similar body heights.

**Table 2:** Sub-analysis of height-matched participants

	Males (*N* = 14)	Females (*N* = 14)	*p*-value (Hedge’s g)
Age (yrs)	29 ± 10	30 ± 13	0.788 (0.086)
Height (m)	1.72 ± 0.03	1.70 ± 0.05	0.088 (0.485)
BMI (kg/m ^2^)	27 ± 4	24 ± 4	0.117 (0.750)
Body fat (%)	22 ± 6	30 ± 8	**0.009 (1.131)**
Resting HR (bpm)	64 ± 17	68 ± 13	0.496 (0.264)
Seated brachial SBP (mmHg)	122 ± 18	117 ± 9	0.415 (0.351)
Seated brachial DBP (mmHg)	75 ± 12	73 ± 7	0.481 (0.204)
Supine brachial SBP (mmHg)	124 ± 9	116 ± 11	0.052 (0.796)
Supine brachial DBP (mmHg)	71 ± 6	70 ± 7	0.892 (0.153)
Aortic SBP (mmHg)	110 ± 6	104 ± 10	0.083 (0.728)
Aortic DBP (mmHg)	72 ± 6	71 ± 7	0.782 (0.153)
MAP (mmHg)	85 ± 7	83 ± 3	0.582 (0.371)
PP (mmHg)	38 ± 6	33 ± 6	**0.034 (0.833)**
AP (mmHg)	5 ± 3	6 ± 3	0.515 (0.333)
AIx (%)	13 ± 9	16 ± 8	0.286 (0.352)
AIx75 (%)	3 ± 9	9 ± 8	0.132 (0.704)
Forward pressure waveform (mmHg)	28 ± 6	25 ± 4	0.244 (0.588)
Backward pressure waveform (mmHg)	14 ± 3	14 ± 3	0.469 (0.000)
Reflection magnitude (%)	53 ± 7	53 ± 4	0.857 (0.000)
PWV (ms ^−1^)	6.08 ± 0.58	5.45 ± 0.59	**0.011 (1.080)**

* Means ± SD*

*HR* heart rate, *SBP* systolic blood pressure, *DBP* diastolic blood pressure, *BMI* body mass index, *MAP* mean arterial pressure, *PP* pulse pressure, *AP* augmentation pressure, *AIx* augmentation index, *AIx75* augmentation index corrected for HR of 75 beats per minute, *PWV* pulse wave velocity

### Central arterial stiffness

**Fig. 4:**
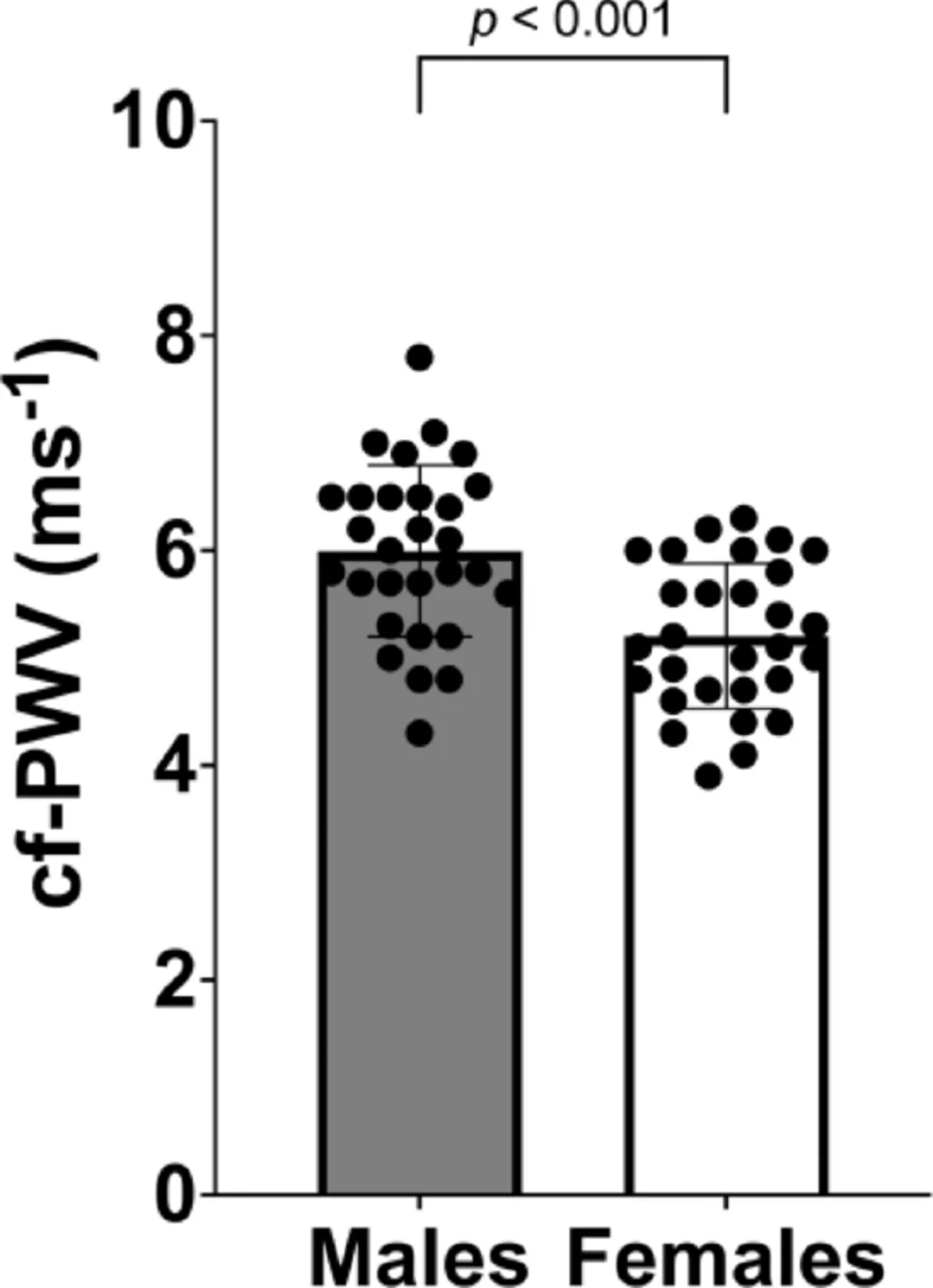
Arterial stiffness was assessed in *N* = 30 males and *N* = 29 females. Carotid-femoral pulse wave velocity was significantly higher in males vs. females before and after covarying for body fat % and age (*p* < 0.001 for both)

Cf-PWV was not reliably obtained from one individual. Therefore, results are presented from *N* = 30 males and *N* = 29 females. Cf-PWV was significantly higher in males vs. females (*p* < 0.001; *g* = 1.092; Fig. 4). Cf-PWV was significantly correlated with total body fat (*r* = −0.315, *p* = 0.015) and age (*r* = 0.383, *p* = 0.003). Linear regression indicated that both body fat (*p* = 0.032) and age (*p* = 0.006) were significant predictors of cf-PWV. When body fat (η_p_^2^ = 0.010) and age (η_p_^2^ = 0.197) were included as covariates, cf-PWV remained significantly higher in males vs. females (*p* < 0.001). cf-PWV was also analyzed in the subset of individuals with similar body height, and remained significantly higher in males compared with females (Table 2, *p* = 0.011, *g* = 1.08).

### Digital thermal monitoring

**Fig. 5:**
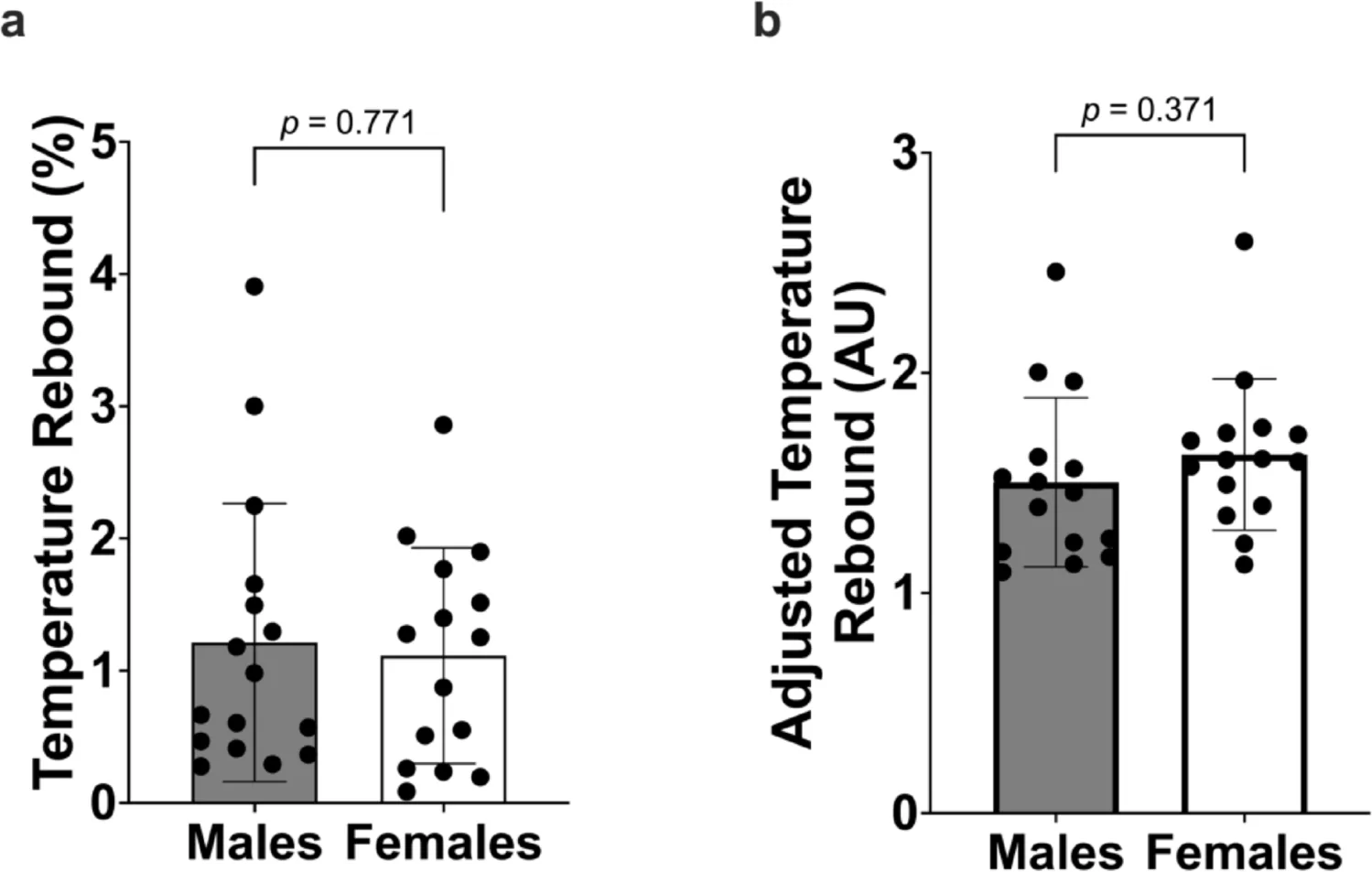
Peripheral vascular reactivity was assessed using digital thermal monitoring during a vascular occlusion test in *N* = 16 males and *N* = 15 females. Sex differences were not observed for **a** temperature rebound (*p* = 0.771) or **b** adjusted temperature rebound (*p* = 0.371)

Fluctuating room temperature (*N* = 5), equipment errors (*N* = 7), sympathetic responses (*N* = 3), and cold fingertips (*N* = 15) led to several participants being excluded from the DTM analyses. A subset of 31 individuals (*N* = 16 male, *N* = 15 female) with reliable data was examined (participant characteristics can be found in Table 3). Baseline temperature was similar between sexes (32.3 ± 0.73 and 32.6 ± 1.28 °C for males and females, respectively; *p* = 0.375). Maximal temperature was also similar between sexes (32.6 ± 0.86 and 32.9 ± 1.23 °C in males and females, respectively; *p* = 0.421. We found no significant differences between males and females in TR (*p* = 0.771, *g* = 0.106; Fig. 5a) or aTR (*p* = 0.371, *g* = 0.327; Fig. 5b). We found no correlations between TR, aTR, and any potential covariates, including age (*r* = −0.067, *p* = 0.719 for TR, and *r* = 0.047, *p* = 0.803).

**Table 3:** Participant characteristics digital thermal monitoring

	Male (*N* = 16)	Female (*N* = 15)	*p*-value (Hedge’s g)
Age (yrs)	27 ± 10	25 ± 9	0.612 (0.209)
Seated resting HR (bpm)	65 ± 16	71 ± 9	0.257 (0.458)
Brachial SBP (mmHg)	119 ± 18	117 ± 8	0.597 (0.142)
Brachial DBP (mmHg)	74 ± 10	73 ± 9	0.615 (0.105)
BMI (kg/m ^2^)	25 ± 4	24 ± 3	0.274 (0.281)
Body Fat (%)	21 ± 6	29 ± 7	**0.004 (2.614)**
ATT (cm)	0.28 ± 0.12	0.39 ± 0.15	**0.018 (0.813)**
MT (cm)	4.29 ± 0.47	3.38 ± 0.49	**< 0.001 (1.897)**
Aerobic exercise (min/wk)	381 ± 247	261 ± 192	0.118 (0.540)
Resistance exercise (min/wk)	202 ± 170	139 ± 103	0.176 (0.445)
Recreational exercise (min/wk)	57 ± 144	69 ± 143	0.512 (0.084)

Means ± SD

*HR* heart rate, *SBP* systolic blood pressure, *DBP* diastolic blood pressure, *BMI* body mass index, *ATT* adipose tissue thickness, *MT* muscle thickness

### Skeletal muscle microvascular reactivity

Table 4 depicts the StO_2_ vascular occlusion test parameters. Baseline StO_2_ was not different between sexes. StO_2_ min was significantly lower in males, while StO_2_ max was similar between sexes. StO_2_ range was significantly greater in males, as was StO_2_ desaturation magnitude. StO_2_ desaturation rate (slope 1) was not significantly different between sexes.

**Table 4:** Near-infrared spectroscopy vascular occlusion test parameters

	Males (*N* = 30)	Females (*N* = 30)	*p*-value (Hedge’s g)
Baseline StO_2_ (%)	64.7 ± 3.6	65.9 ± 3.8	0.207 (0.325)
StO_2_ Min (%)	36.5 ± 8.0	43.8 ± 8.2*	0.001 (0.898)
StO_2_ Max (%)	77.4 ± 2.4	76.2 ± 2.5	0.069 (0.478)
StO_2_ Desaturation Magnitude (%)	28.2 ± 7.1	22.2 ± 7.0*	0.002 (0.848)
StO_2_ Range (AU)	40.9 ± 8	32.4 ± 9.7*	0.001 (0.954)
StO_2_ Slope 1 (%s^− 1^)	−0.11 ± 0.02	−0.12 ± 0.15	0.809 (0.064)

StO_2_, tissue oxygen saturation; *AU* arbitrary units, Means ± SD

*indicates statistical difference at *P* < 0.05

**Fig. 6:**
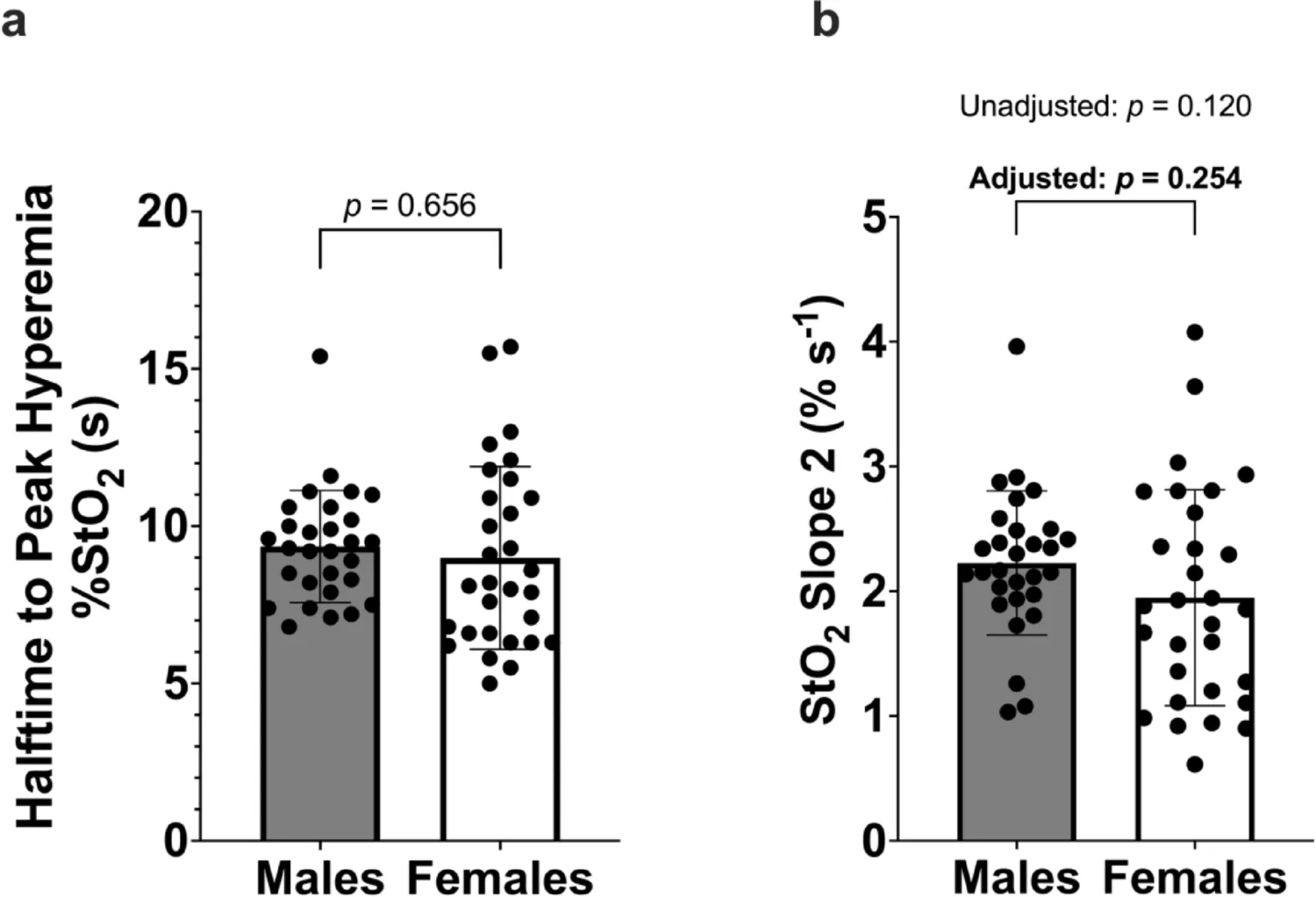
Microvascular reactivity was assessed using near-infrared spectroscopy during a vascular occlusion test in *N* = 30 males and *N* = 30 females. Sex differences were not observed for **a** StO_2_ halftime to peak hyperemia (*p* = 0.656) or **b** StO_2_ slope 2 (*p* = 0.120), even after covarying for desaturation magnitude and min/week of resistance training (*p* = 0.254)

When examining microvascular reactivity, we observed no sex differences between the StO_2_ T ½ (*p* = 0.656, *g* = 0.116; Fig. 6a) or the StO_2_ slope 2 (*p* = 0.121, *g* = 0.406; Fig. 6b). StO_2_ T ½ was not correlated with any potential covariates. Similar to our previous study (Landers-Ramos et al. 2024a), we found that StO_2_ slope 2 was significantly correlated with ATT (*r* = −0.433, *p* = 0.001), along with body fat (*r* = −0.337, *p* = 0.008), desaturation magnitude (*r* = 0.558, *p* < 0.001), and minutes per week of RT (*r* = 0.316, *p* = 0.014). We found no relationship between StO_2_ slope 2 and age (*r* = 0.054, *p* = 0.680). Linear regression indicated that desaturation magnitude (*p* < 0.001) and minutes per week of RT (*p* = 0.038) were the only significant predictors of StO_2_ slope 2. When desaturation magnitude (η_p_^2^ = 0.303) and minutes per week of RT (η_p_^2^ = 0.094) were included in an ANCOVA, our original findings remained (*p* = 0.254). Although not determined in this study to be significant predictors of StO_2_ slope 2, we explored other covariates recently determined to have a significant impact on StO_2_ slope 2 when examining sex differences (Murias et al. 2026). Specifically, we performed ANCOVAs covarying for desaturation rate (η_p_^2^ = 0.012) and magnitude (η_p_^2^ = 0.259) or desaturation rate (η_p_^2^ = 0.011), desaturation magnitude (η_p_^2^ = 0.154), and ATT (η_p_^2^ = 0.016), and continued to see no sex differences in StO_2_ slope 2 when performing either analysis (*p* = 0.918 and *p* = 0.684, respectively).

### Correlations between vascular outcomes

Cf-PWV was significantly correlated with AIx75 (*r* = −0.329, *p* = 0.012) but not AIx (*r* = −0.255, *p* = 0.056), forward pressure waveform (*r* = −0.106, *p* = 0.433), backward pressure waveform (*r* = −0.184, *p* = 0.171), or reflection magnitude (*r* = −0.102, *p* = 0.452).

StO_2_ T ½ was significantly correlated with StO_2_ slope 2 (*r* = −0.599, *p* < 0.001). StO_2_ slope 2 was not correlated with StO_2_ slope 1 (*r* = −0.197, *p* = 0.132). We found no significant relationship between StO_2_ slope 2 and TR (*r* = −0.300, *p* = 0.099) or aTR (*r* = 0.319, *p* = 0.081).

## Discussion

Currently, the effect of biological sex on different vascular outcomes is incompletely understood. In this study of recreationally active, healthy adults, we found that there were no sex differences in AIx when accounting for body height, while arterial stiffness (cf-PWV) was more favorable in females compared with males. We also found no sex differences in forearm muscle microvascular reactivity or in peripheral vascular reactivity. Finally, in an exploratory analysis, we found no significant relationship between NIRS-based microvascular reactivity and DTM-based peripheral vascular reactivity. Collectively, after accounting for confounding variables, differences in cf-PWV were detected between sexes, while sex differences in other assessments of vascular health were not observed.

Studies demonstrating higher AIx in females across adults of all ages have been previously published (McEniery et al. 2005), and this has held even after adjustment for age, HR, and MAP (Lieber et al. 2010; Shim et al. 2011). However, in previous literature, adjustments for body height are often not accounted for. A smaller body stature, as commonly exhibited by females, is believed to cause an earlier wave reflection and therefore, a higher AIx (Doonan et al. 2013). In the present study, sex differences in AIx and AIx75 were no longer evident after covarying for body height. These findings are consistent with others (Lieber et al. 2010; Shim et al. 2011; Doonan et al. 2013; Landers-Ramos et al. 2022) demonstrating an impactful contribution of body height on AIx. Still, one study that we are aware of found that sex differences persisted when males and females were matched for height (Gatzka et al. 2001). This suggests that wave reflection differences between sexes extend beyond differences in body stature and potentially reflect other structural differences, such as increased tapering of the arterial diameter from the aorta to the periphery in females (Cecelja et al. 2009). However, it should also be noted that the participants in the Gatzka et al. study were elderly, suggesting sex differences in augmentation index in older adults (Gatzka et al. 2001; Zaydun et al. 2006) that may not be present in younger and middle-aged adults. To examine this idea further in our current study of premenopausal females and similarly aged males, we performed analyses on a subset of individuals with similar body height and found no sex differences in AIx or AIx75. Our sample size in this subset analysis was on the lower end of what we calculated to elicit appropriate statistical power. However, these results, paired with our results from the full cohort adjusted for body height, suggest that body stature is a main contributor to sex differences in AIx and should be accounted for to best interpret true vascular properties in males vs. females.

Analyses of forward and backward pressure waveforms may provide additional information to aid in the interpretation of AIx findings (Torjesen et al. 2014). In this study, we found that the forward pressure waveform was significantly higher in males, whereas the backward pressure waveform and reflection magnitude did not differ between sexes. The higher forward pressure waveform is indicative of a greater ventricular contraction and PWV of the proximal aorta (Butlin and Qasem 2016; Li et al. 2017), and is likely explained by a larger heart size in males. In contrast, the backward pressure waveform is believed to be influenced by the stiffness of the peripheral vasculature at major sites of reflection (Butlin and Qasem 2016; Li et al. 2017). Further, sex differences in AP were eliminated when covarying for body height or when comparing in a subset of individuals matched for body height, while lower PP in females remained. Thus, dissecting the different components of wave reflection provides further support for the AIx results likely being influenced by body height and not necessarily being indicative of differences in vascular stiffening. Importantly, cf-PWV is currently the reference standard measure for arterial stiffness (Tanaka 2018). Even after covarying for age and body fat %, we found cf-PWV to be lower in females compared with males. This is despite a significant relationship between cf-PWV and AIx75. Lower cf-PWV in females has been previously reported in the literature (Waddell et al. 2001; Reference Values for Arterial Stiffness Collaboration 2010) and is consistent with reduced PP in females, a widely used rudimentary indicator of arterial stiffness (Mitchell et al. 2004). However, it should be noted that the average cf-PWV for all individuals fell at or below normative values for all ages included in this investigation (Diaz et al. 2018). Thus, it is currently unclear whether the observed sex differences in cf-PWV are indicative of greater CVD risk later in life. Collectively, when we look at the different outcomes assessing components of arterial stiffness, we find that these demonstrate similar or slightly lower arterial stiffness in premenopausal females compared with males, which is consistent with global CVD risk statistics (Benjamin et al. 2018; Stanhewicz et al. 2018).

Vascular reactivity in response to hyperemia is used to assess endothelial function (Limberg et al. 2020; Murias et al. 2026). Many techniques have been used to non-invasively assess endothelial function, each with conflicting evidence regarding sex differences (Zocalo and Bia 2021; Fekete et al. 2025). In this study, we selected measures of microvascular reactivity as they may provide an earlier indicator of vascular dysfunction in younger to middle-aged adults (Limberg et al. 2020). We found no significant differences between sexes when examining NIRS-derived slope 2 or T ½. Sex differences in NIRS-derived measures of microvascular reactivity have been commonly reported, with males typically (Rasica et al. 2022; Keller et al. 2023; Murias et al. 2026) but not always (Zaleski et al. 2023) exhibiting greater microvascular reactivity. ATT is known to have a substantial confounding influence on NIRS signals (van Beekvelt et al. 2001; Landers-Ramos et al. 2024a), with a recent study by van Beekvelt et al. indicating that ATT explained nearly 54% of the variance in NIRS outcomes (van Beekvelt et al. 2025). Thus, recent efforts have been made to understand the contributing effects of ATT on NIRS-derived microvascular reactivity (Keller et al. 2023; Landers-Ramos et al. 2024a; Murias et al. 2026). Interestingly, in the present study, only 18.7% of the variation in StO2 slope 2 was explained by ATT. While this was higher in the females (28.9% vs. 0.5% in males), our regression analysis revealed that ATT was not a significant predictor of StO2 slope 2. A recent study by Murias et al. examined several statistical approaches to account for potential sex differences and concluded that using ANCOVA to account for differences in StO_2_ desaturation rate and StO_2_ magnitude may provide a more accurate approach for examining microvascular reactivity in males vs. females (Murias et al. 2026). When we performed an exploratory ANCOVA to determine whether the approach taken by Murias et al. would apply to our findings, we continued to see no sex differences in forearm muscle microvascular reactivity. These findings are aligned with conclusions from a recent meta-analysis including 14 articles (Fekete et al. 2025) assessing other measures of post-occlusion reactive hyperemia (ultrasound Doppler and skin Laser Doppler).

Our NIRS-based microvascular reactivity findings may be affected by the muscle studied. Lower limb muscles are more frequently studied in the literature (Rasica et al. 2022; Keller et al. 2023; Citherlet et al. 2024; Landers-Ramos et al. 2024a; Murias et al. 2026). However, differences in physical activity between sexes may influence microvascular health (Rasica et al. 2022). In the present study, we chose to examine a non-ambulatory limb that may be less influenced by physical activity. Notably, we found that time spent in recreational sports and aerobic activity was similar between sexes. While males did report more time performing RT, including RT as a covariate did not alter our results. Our findings are supported by others reporting no sex differences in forearm microvascular reactivity (Jeskanen et al. 2024; Perlet et al. 2024). One study examining the forearm muscle found that sex was not a significant factor in their analyses of slope 2 responses to RT, while greater slope 2 values were observed in males in the vastus lateralis muscle (Perlet et al. 2024). Thus, the presence of sex differences may be dependent on the muscle examined. Still, at least one other study found that females have greater reperfusion in both the forearm and the quadriceps (Zaleski et al. 2023), warranting more research in this area for a complete understanding of muscle-specific sex differences in NIRS-based microvascular reactivity.

We simultaneously used DTM along with NIRS to obtain more information on microvascular function in males vs. females. We found no sex differences in our DTM measures of TR or aTR. These findings align with at least one other study examining temperature rebound with DTM in 26 patients (*N* = 17 male) (Schier et al. 2014). Still, other studies have reported that the percentage of “good” vascular reactivity index, as assessed by DTM, is higher in females in a large study consisting of over 6000 patients (Naghavi et al. 2016, 2022), whereas “poor” vascular reactivity index has been reportedly not different between sexes (Naghavi et al. 2022) or slightly lower in males (Naghavi et al. 2016). Most literature regarding DTM relates to its clinical utility and relationship with other CVD risk factors (Ahmadi et al. 2008, 2009; Frolow et al. 2015; Naghavi et al. 2016, 2022; Frech and Murtaugh 2019). Thus, the topic has not been extensively studied in active healthy populations for comparison. For our DTM outcome, our sample size was reduced due to approximately half of the tests being excluded because of fluctuating room temperature, equipment errors, sympathetic responses, and cold fingertips. This may limit the interpretability of our results. Furthermore, females tend to have ~ 2 degrees lower hand temperature than males while having similar core temperatures (Kim et al. 1998), potentially leading to additional bias when using DTM. In the participants tested, baseline finger temperature was similar between sexes, and initial finger temperature was accounted for in the temperature rebound calculations. However, while finger temperature differences may not have affected our present findings, 60% of the participants excluded from the DTM tests due to cold fingers were female. Thus, the number of participants eligible for testing should be considered when attempting to draw comparisons using this method. Collectively, further studies using DTM are necessary for a more complete understanding of sex differences in this outcome, particularly in younger healthy adults, as it may reveal early CVD risk and expand upon the clinical feasibility of this tool with advancing age.

In an exploratory analysis, we found no relationship between our NIRS-based microvascular reactivity and DTM-derived TR or aTR. While this is contrary to our hypothesis, these findings align with previous studies that have assessed DTM-derived outcomes and other microvascular measures (Dhindsa et al. 2008; Frolow et al. 2015). DTM is considered both a macro- and micro-vascular test (Akhtar et al. 2010) but is predominantly thought to assess microvascular responses (Schier et al. 2014). Interestingly, moderate but significant relationships between DTM outcomes and macrovascular function are more commonly reported (Dhindsa et al. 2008; Akhtar et al. 2010; Frech and Murtaugh 2019; Heath et al. 2021), suggesting a potentially greater macrovascular contribution in this measure than previously credited. Still, a lack of relationship between similar vascular assessments is not uncommon, with one study finding that different vascular reactivity measures were not significantly associated in more than 75% of cases (Dhindsa et al. 2008). This is likely because each measure assesses a unique aspect of vascular function that does not completely equate. Thus, examining multiple parameters of vascular reactivity paints a more complete picture of the varying physiological mechanisms underlying peripheral vascular responses and allows for a better understanding of potential differences between sexes.

One potential limitation in the present study was that the occlusion cuff pressure during the simultaneous microvascular assessments was individualized based on 50 mmHg above SBP. This protocol was based on the automated features of DTM equipment, is standard practice for DTM, and is in accordance with recommendations for VOTs (Barstow 2019). However, many NIRS-based protocols typically perform occlusion at 200–250 mmHg for the upper body (Zaleski et al. 2023; Greaves et al. 2024; Jeskanen et al. 2024; Perlet et al. 2024). Thus, direct comparisons with other studies may be limited. Another limitation of the current study was that we grouped younger and middle-aged individuals. We opted to do this to reduce the influence of accelerated vascular aging after menopause in females (Stanhewicz et al. 2018). While the number of younger and middle-aged individuals was similar in the male and female groups, we did not account for the effects of perimenopause in our female participants, which may affect some vascular outcomes (Hildreth et al. 2018; Dubose and Lefferts 2023). Further research is needed on the effects of menopausal transition on vascular health in females and how this compares with similarly aged males. Additionally, physical activity levels can impact vascular outcomes, and it is possible that our findings in this population of recreationally active individuals may not be generalizable to inactive or highly trained individuals. Finally, while we hope the results from this study can inform future CVD risk, it is unclear whether the sex differences we observed in this study are associated with CVD risk seen later in life.

In conclusion, differences in three aspects of vascular health (AIx, DTM-based vascular reactivity, and microvascular reactivity) were not detected based on biological sex in recreationally active younger and middle-aged adults. Small, but significant, elevations in cf-PWV were observed in males compared with females, although all values fell within or below the normative range for this age group (Diaz et al. 2018). Still, even small differences can aid in more individualized monitoring and treatment of CVD, particularly if greater risk can be identified in younger or middle-aged individuals. Collectively, these findings help clarify which vascular indices differ between males and females and highlight the influence of anthropometric factors, such as body height, on commonly used measures of wave reflection.

## Data Availability

Raw data are available upon reasonable request.

## List of symbols

AIx: Augmentation index
AIx75: Augmentation index normalized to 75 bpm
ANCOVA: Analysis of covariance
AP: Augmentation pressure
aTR: Adjusted temperature rebound
ATT: Adipose tissue thickness
AU: Arbitrary unit
BMI: Body mass index
Cf-PWV: Carotid-femoral pulse wave velocity
CVD: Cardiovascular disease
DBP: Diastolic blood pressure
DTM: Digital thermal monitoring
DXA: Dual-energy X-ray absorptiometry
HHb: Deoxygenated hemoglobin
O_2_Hb: Oxygenated hemoglobin
HR: Heart rate
MAP: Mean arterial pressure
MT: Muscle thickness
mVO_2_: Muscle oxygen consumption
NIRS: Near-infrared spectroscopy
O.D: Optical density
Pb: Reflected pressure waveform
Pf: Forward pressure waveform
PP: Pulse pressure
RT: Resistance training
SBP: Systolic blood pressure
SD: Standard deviation
StO_2_: Tissue oxygen saturation
T ½: Halftime to peak hyperemia
TR: Temperature rebound
VOT: Vascular occlusion test
